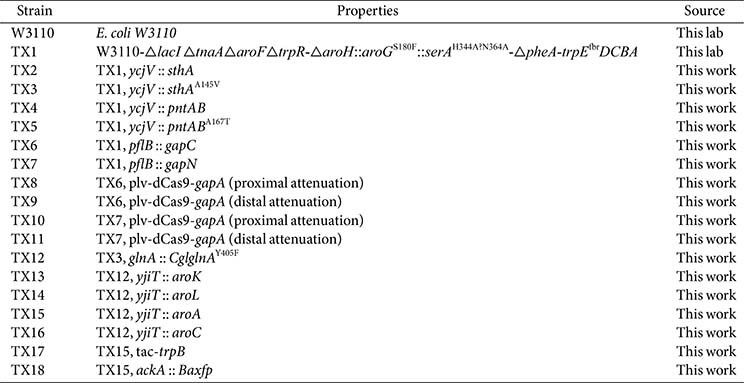# Optimizing L-Tryptophan Production in *Escherichia coli* through Redox Balancing and Metabolomics Analysis

**DOI:** 10.4014/jmb.2508.08025

**Published:** 2025-12-29

**Authors:** Tongxin Wan, Dongqin Ding, Junqing Chen, Yaru Zhu, Huiying Wang, Zixiang Xu, Junlin Yang, Yufeng Wang, Jia Song, Dawei Zhang

**Affiliations:** 1 College of Biotechnology, Tianjin University of Science & Technology, Tianjin 300457, P.R. China; 2 Tianjin Institute of Industrial Biotechnology, Chinese Academy of Sciences, Tianjin 300308, P.R. China; 3 University of Chinese Academy of Sciences, Beijing 100049, P.R. China; 4 State Key Laboratory of Engineering Biology for Low-Carbon Manufacturing, Tianjin Institute of Industrial Biotechnology, Chinese Academy of Sciences, Tianjin 300308, P.R. China; 5 National Center of Technology Innovation for Synthetic Biology, Tianjin 300308, P.R. China; 6 Dalian Polytechnic University, Dalian, 116000, P.R. China; 7 Yuxing Biotechnology (GROUP) CO., LTD., P.R. China

**Keywords:** *Escherichia coli*, redox engineering, metabolomics analysis, fermentation optimization

## Abstract

L-tryptophan (L-trp) is a key aromatic amino acid with significant industrial value, and microbial fermentation provides a sustainable alternative to traditional chemical synthesis. However, low production yields due to inefficient microbial strains remain a major challenge. In this study, we enhanced L-trp production through redox engineering of *Escherichia coli* TX1. Metabolomics analysis at various fermentation stages revealed dynamic changes in the metabolites of the aromatic amino acid pathway. A key bottleneck was identified in the shikimate pathway, where significant accumulation of chorismate and shikimate led to inefficient L-trp production. By optimizing the shikimate pathway, L-trp production was increased by 19.8%. Additionally, the continuous accumulation of phosphoenolpyruvate suggested a limitation in the supply of erythrose-4-phosphate, which participates in the same reaction. Redirecting carbon flux from fructose-6-phosphate toward erythrose-4-phosphate increased the precursor pool of erythrose-4-phosphate. To overcome nutritional limitations, exogenous addition of amino acids, vitamins, and salt ions to the fermentation medium was implemented. Systematic metabolic engineering and fermentation optimization led to a significant improvement in tryptophan production, achieving an 86.6% increase compared to the original level. This study lays a solid foundation for the future development of more efficient tryptophan-producing strains.

## Introduction

L-Trp is an essential aromatic amino acid with a wide range of applications in nutrition, endocrine regulation, and pharmaceuticals [1-3]. The global demand for L-trp is increasing annually. Traditional production methods, such as casein hydrolysis and chemical synthesis, are complex, polluting, and costly. Enzyme and microbial conversion methods, driven by biotechnology, have limitations due to high raw material costs and poor enzyme stability. In contrast, microbial fermentation using engineered strains of *E. coli* or *Corynebacterium glutamicum* is now recognized as the most promising technology for L-trp production [4-6], This method directly ferments inexpensive carbon sources like glucose (Glc), offering low raw material costs, environmentally friendly processes, high cell density, and high production efficiency. With the continuous development of synthetic biology, rational metabolic engineering plays a key role in breaking through the limitations of the inherent metabolic networks in wild-type strains. By rewiring metabolic fluxes [7], alleviating feedback inhibition [8], enhancing enzyme activity [9], optimizing precursor availability [10] and transport systems [11], and eliminating competing pathways through systematic gene deletions [12], as well as coupling strain growth with tryptophan production [13], high-performance microbial cell factories can be developed. These factories are capable of producing L-trp with high titers, yields, and productivities in an efficient and sustainable manner, thus meeting the ever-increasing market demand.

**Fig. 1:**
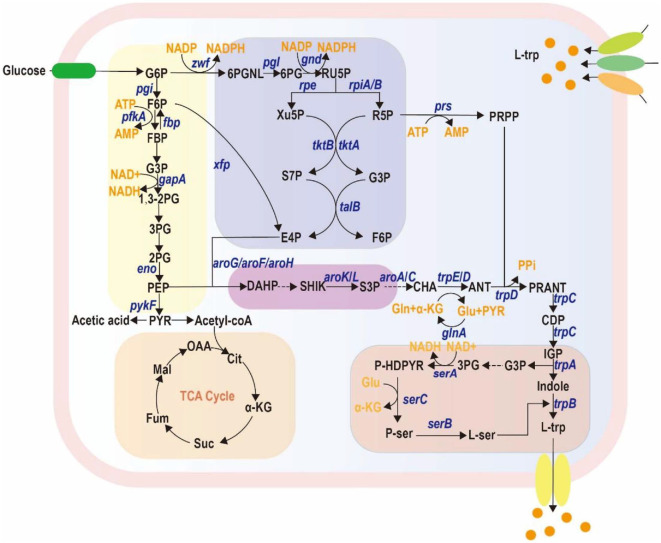
The biosynthetic pathway of tryptophan from glucose. Abbreviations: *pgi*, glucose-6-phosphate isomerase; *zwf*, glucose-6-phosphate dehydrogenase; *pfkA*, phosphofructokinase-1; *fbp*, fructose-bisphosphatase; *gapA*, glyceraldehyde-3-phosphate dehydrogenase; *eno*, enolase; *pykF*, pyruvate kinase; *gnd*, gluconate dehydrogenase; *rpe*, ribulose-phosphate 3-epimerase; *rpiA*, ribose-5-phosphate isomerase; *prs*, phosphoribosyl synthetase; *tktA*, transketolase; *tktB*, transketolase; *talB*, transaldolase; *aroG*, 3-dehydroquinate synthase; *trpE/D*, anthranilate synthase; *glnA*, glutamine synthetase; *trpC*, indole-3-glycerol phosphate synthase; *trpA*, tryptophan synthase alpha subunit; *trpB*, tryptophan synthase beta subunit; *serA*, phosphoglycerate dehydrogenase; *serC*, hydroxymethyltransferase; *serB*, phosphoserine phosphatase; G6P, lucose-6-phosphate; 6PGNL, 6-phosphogluconolactone; 6PG, 6-phosphogluconate; RU5P, ribulose-5-phosphate; F6P, fructose-6-phosphate; FBP, fructose-1,6-bisphosphate; G3P, glyceraldehyde-3-phosphate; 3PG, 3-phosphoglycerate; 2PG, 2-phosphoglycerate; PEP, phosphoenolpyruvate; PYR, pyruvate; OAA, oxaloacetate; SuC, succinate; α-KG, α-ketoglutarate; AccoA, acetyl-coA; Cit, Citrate; Mal, Malate; Fum, Fumarate; X5P, xylulose-5-phosphate; R5P, ribose-5-phosphate; PRPP, 5-phospho-α-D-ribose 1-diphosphate; S7P, sedoheptulose-7-phosphate; E4P, erythrose-4-phosphate; DAHP, 3-deoxy-Darabino-heptulosonate 7-phosphate; SHIK , shikimate; CHA, chorismate; ANT, anthranilate; PRANT, N-(5-phosphoribosyl)-anthranilate; CDP, (1S,2R)-1-C-(indol-3-yl)glycerol 3-phosphate; IGP, 1-(2-carboxyphenylamino)-1-deoxy-D-ribulose 5-phosphate; P-HDPYR, 3-phosphooxypyruvate; P-ser, phosphoserine; L-ser, L-serine; L-trp, L-tryptophan; Gln, glutamine; Glu, glutamate.

The synthesis of L-trp mainly involves the generation of phosphoenolpyruvate (PEP) through glycolysis and synthesis of erythrose-4-phosphate (E4P) through the pentose phosphate pathway (Fig. 1) [6, 20]. These two precursors are then converted to 3-deoxy-D-arabino-heptulosonate 7-phosphate (DAHP), which enters the shikimate pathway and ultimately leads to the synthesis of L-trp. Additionally, the biosynthesis of L-trp requires L-serine, glutamine, and 5-phosphoribosyl-1-pyrophosphate (PRPP) as precursors, which are derived from the serine biosynthesis pathway, the tricarboxylric acid (TCA) cycle, and the pentose phosphate pathway, respectively [6]. The requirement for multiple precursors defines the complexity of the L-trp biosynthetic pathway. Balancing the metabolic fluxes of glycolysis, the pentose phosphate pathway, the serine biosynthesis pathway, and the TCA cycle is crucial for enhancing L-trp production. Specifically, optimizing the allocation of carbon and energy resources among these pathways can significantly impact the availability of key precursors such as PEP, E4P, and PRPP. For instance, redirecting flux from competing pathways, such as those involved in the synthesis of other amino acids or secondary metabolites, can help channel more resources towards L-trp production. Additionally, fine-tuning the expression of enzymes involved in these pathways, either through genetic engineering or regulatory modifications, can further enhance precursor availability and overall yield.

Within the tryptophan biosynthetic pathway, dynamic changes in reducing power can influence the redox state of metabolic intermediates, thereby significantly impacting the efficiency of tryptophan synthesis. Cofactor regulation is crucial for constructing efficient cell factories [21, 22]. Over the past few decades, research has primarily focused on the metabolic regulation of NADH and NADPH. The interconversion between NADH and NADPH is regulated by soluble transhydrogenase (SthA) and membrane-bound transhydrogenase (PntAB) [24]. The *sthA* gene encodes soluble pyridine nucleotide transhydrogenase, a non-energy-dependent flavoprotein that exists as a soluble multimer. It catalyzes the hydrogen transfer reaction between NADH and NADP^+^, thereby transferring the reducing power of NADH to NADP^+^ to generate NADPH. This process is crucial for maintaining cellular redox balance and promoting biosynthesis. The *pntAB* gene encodes membrane-bound pyridine nucleotide transhydrogenase, composed of two subunits, and relies on proton motive force to drive the hydrogen transfer between NADH and NADP^+^. Both enzymes play key roles in the regeneration of NADPH and metabolic regulation within the cell. Research has shown that overexpression of the *pntAB* or *sthA* genes significantly boosts the regeneration of NADPH. This approach has been effectively applied in the metabolic engineering of amino acids like shikimic acid [25], 3-hydroxypropionic acid [26] 1,4-diaminobutane [23] L-arginine [27], and L-lysine [28], leading to enhanced yields of the target metabolites.

In this study, a well-characterized *E. coli* K-12 W3110 strain was developed for L-trp production through rational metabolic engineering (Table 1). Key strategies included enhancing the supply of NADPH, pinpointing metabolic bottlenecks via metabolomics analysis, and optimizing the fermentation medium. The introduction of the SthA mutant effectively increased the supply of NADPH, thereby boosting L-trp production. Through omics analysis, it was precisely determined that the shikimate pathway and the limited supply of E4P were key factors restricting further increases in L-trp production. Additionally, the addition of threonine met the changing nutritional demands of the strain during the engineering process. Ultimately, the engineered strain TX18 achieved a L-trp production of 4.57 g/l, an 86.6% increase compared to the initial strain, laying a solid foundation for the development of subsequent tryptophan-producing strains.

## Materials and Methods

### Strains and Plasmids

*E. coli* DH5α was used for plasmid construction, whereas *E. coli* TX1 was selected as the chassis strain for L-trp production. Details regarding the strains, primers in this study are provided in the Tables 1 and S1.

### Fermentation in Shake Flasks

A 250 ml shake flask containing 19 ml of fermentation medium was inoculated with seed culture grown in 1 ml of Luria-Bertani medium (LB) (OD_600_ ≥ 2.0). Incubation was performed at 37°C and 220 rpm for 38 h. The fermentation medium contained 30 g/l glucose, 0.5 g/l MgSO_4_·7H_2_O, 3 g/l KH_2_PO_4_, 12 g/l K_2_HPO_4_, 4 g/l (NH_4_)_2_SO_4_, 1 g/l yeast extract, 0.1 g/l sodium citrate, 0.1 g/l FeSO_4_·7H_2_O, pH adjusted to 7.0 with NaOH.

### Analysis

**OD_600_ measurement.** Cell growth was measured by monitoring optical density at 600 nm using a spectrophotometer. The bacterial fermentation broth was diluted with deionized water, and the optical density at 600 nm (OD_600_) was measured. The OD_600_ value after dilution should ideally fall within the range of 0.2 to 0.8. A V-1600 visible spectrophotometer (Shanghai Meipuda Instrument Co., Ltd., China) was used.

**Liquid chromatography analysis.** A 1 ml sample of the fermentation broth was centrifuged at 13,000 rpm for 3 min. The supernatant was collected and diluted for high-performance liquid chromatography (HPLC) analysis of L-trp and glucose concentrations. Before HPLC analysis, the supernatant was filtered through a 0.22 μm pore size membrane. The L-trp content was determined using a Zorbax Eclipse AAA column. The mobile phase consisted of solvent A (40 mM NaH_2_PO_4_, pH adjusted to 7.8 with NaOH) and solvent B (ACN: MeOH: water = 9:9:2, v/v/v), with a flow rate of 2 ml/min. During injection, the ratio of solvents A and B was as follows: 100% A (0-1.9 min), 43% A and 57% B (1.9-18.1 min), 100% B (18.1-22.3 min), and 100% A (22.3-26 min). The retention time of L-trp was 12.9 min, and the detection wavelength was UV 338 nm [29].

### Metabolomics Analysis

During the 250 ml shake flask fermentation process, samples were collected at 6 h, 18 h, and 30 h for untargeted quantitative metabolomics analysis (Additional file 1: Fig. S1). The culture with the same optical density was subjected to low-temperature centrifugation at 4°C, 8,000 rpm for 5 min. The supernatant was discarded, and the pellet was resuspended in 5 ml of PBS solution for washing. The washing step was repeated twice. The resulting cell pellet was immediately frozen in liquid nitrogen for 15 min and then stored at -80°C. Each sample sent for analysis had a biomass weight of ≥ 0.2 g. The samples were taken out from -80°C and then 0.5 ml of methanol, acetonitrile, and water solution (2:2:1, v/v) was added respectively. The mixture was vortexed for 60 s, sonicated at low temperature for 30 min (twice), and then placed at -20°C for 1 h to precipitate proteins. After that, the samples were centrifuged at 14,000 rcf for 20 min at 4°C. The supernatant was collected for lyophilization and the samples were stored at -80°C.

Intracellular metabolites were analyzed using an ultra-high-performance liquid chromatography (UHPLC) system (Agilent 1290 Infinity LC, USA) coupled with a mass spectrometer (AB TripleTOF 6600, AB Sciex, USA) in negative ion mode under electrospray ionization (ESI). Metabolite separation was performed using an ACQUITY UPLC BEH Amide column (1.7 μm, 2.1 mm × 100 mm, Waters, USA). The column temperature was set to 25°C, with a flow rate of 0.5 mL/min and an injection volume of 2 μL. The mobile phase consisted of A: water+ 25 mM ammonium acetate + 25 mM ammonia solution, and B: acetonitrile. The gradient elution program was as follows: 0-0.5 min, 95% B; 0.5-7 min, B decreased linearly from 95% to 65%; 7-8 min, B decreased linearly from 65% to 40%; 8-9 min, B was maintained at 40%; 9-9.1 min, B increased linearly from 40% to 95%; 9.1-12 min, B was maintained at 95%. Throughout the analysis, the samples were kept at 4°C in an automatic sampler. The relative abundance of metabolites was normalized to cell density.

During data processing, the metabolite levels of the TX3 strain at 6 h were used as the reference standard and set to a value of 1. The metabolite levels of the TX3 strain at 18 h and 30 h, as well as those of the TX15 strain at 6 h, 18 h, and 30 h, were normalized relative to the data of the TX3 strain at 6 h. The resulting ratios reflect the relative levels of each metabolite.

### Method for Plasmid Construction

**Plasmid construction using Cas9-pntAB as an example.** In *E. coli*, endogenous gene knockouts and integrations, as well as exogenous gene integration, were achieved using the CRISPR/Cas9 system [30]. To construct the cas9-*pntAB* plasmid, the *E. coli* K12 W3110 was used as the template. The *pntAB*-up fragment, containing homologous arms, was amplified using primers *ycjV*-up-F and *pntAB*-up-R, while the *pntAB*-down fragment, also with homologous arms, was amplified using primers *pntAB*-down-F and *ycjV*-down-R. Subsequently, an overlap extension PCR was conducted using the *pntAB*-up and *pntAB*-down fragments as templates, with primers *ycjV*-up-F and *ycjV*-down-R, to generate the seamless *pntAB*-UD fragment.

Next, using the cas9 plasmid as the template, two plasmid backbone fragments were amplified: *pntAB*-ver1 using primers *ycjV*-ver-F and *ycjV*-N20-R, and *pntAB*-ver2 using primers *ycjV*-N20-F and *ycjV*-ver-R, both containing homologous arms. These fragments were then assembled together with the *pntAB*-UD fragment via Gibson Assembly to obtain the final cas9-*pntAB* plasmid. All primers used in this study are listed in the supplementary materials.

All cas9 plasmids in this study were constructed following this procedure.

### Principle of dCas9-Mediated Gene Attenuation

CRISPR interference (CRISPRi) technology employs a catalytically inactive Cas mutant (such as dCas9) that, guided by a specific guide RNA (gRNA), binds to the target gene locus and inhibits transcription by blocking RNA polymerase binding [31]. dCas9 suppresses gene expression by either obstructing the RNA polymerase binding site or interfering with transcriptional elongation. In this study, the *gapA* gene was attenuated using this approach, with two strategies: proximal attenuation (targeting the first 100 bp of the *gapA* coding region) and distal attenuation (targeting the 200–300 bp region of the *gapA* coding sequence). The proximal attenuation sequence used in this study is: cctgttagacgctgattaca.

### Quantitative Polymerase Chain Reaction (qPCR) Analysis

Sample Processing: Fermentation was conducted using strains TX6 and TX8. Sampling was collected at 6, 18, and 30 h, with each sample comprising a 1.0 OD_600_ cell suspension. These samples were then stored at -80°C. After confirming normal yield through HPLC, RNA extraction was performed.

RNA Extraction: RNA extraction was performed utilizing the RNA extraction kit from Tiangen. The purity (A_260_/A_280_ ratio) and concentration of the extracted RNA were determined using a NanoDrop spectrophotometer (Thermo Fisher, USA). RNA samples with an A_260_/A_280_ ratio between 1.8 and 2.0, showing no significant degradation or DNA contamination, were considered suitable for further use.

Reverse Transcription to Synthesize cDNA: For reverse transcription, 1 μg of RNA per sample was used. The NovoScript Plus All-in-one 1st Strand cDNA Synthesis SuperMix (gDNA Purge) kit was employed according to the manufacturer's instructions. The total reaction volume was 20 μl, with the reaction conditions being 50°C for 15 min and 85°C for 5 sec. The prepared cDNA samples were stored at -20°C for later use.

qPCR Reaction: All qPCR experiments were conducted on the Roche LightCycler 96. The reaction mixture was prepared as follows: a total volume of 20 μl per reaction, consisting of 10 μl of 2xNovoStart SYBR qPCR SuperMix Plus, 1 μl of forward primer, 1 μl of reverse primer, 1 μl of cDNA template, and sterile deionized water to make up the volume to 20 μl. The qPCR conditions were as follows: initial denaturation at 95°C for 60 s, followed by 40 cycles of 95°C for 20 sec, 60°C for 30 sec, and 72°C for 30 sec. After the reaction, the specificity of the products was confirmed by melting curve analysis, and a no-template negative control was set to ensure the specificity and accuracy of the reaction system.

Data Analysis: The results of qPCR were analyzed by the 2^-ΔΔCt^ method for relative quantification. The difference in Ct values between the target gene and the reference gene was first calculated (ΔCt=Ct value of target gene-Ct value of reference gene). Then the ΔΔCt values for the experimental and control groups were determined (ΔΔCt=Ct value of experimental group-Ct value of control group). The relative expression level was calculated as 2^-ΔΔCt^. The relative expression level was used to evaluate the differences in target gene expression between the experimental and control groups. A data value less than 1 indicated downregulation of the target gene (the target gene was the weakener of the *gapA* gene, and the control group was the *gapA* gene).

### Quantification of Intracellular NADPH Levels

The NADP^+^/NADPH Assay Kit with WST-8 (Beyotime Institute of Biotechnology, China) was employed to determine the levels of NADP^+^ (oxidized form of nicotinamide adenine dinucleotide phosphate) and NADPH (reduced form of nicotinamide adenine dinucleotide phosphate) in cells, tissues or other samples, as well as their respective ratio and total amount. This assay kit is based on the colorimetric reaction of WST-8.

The principle of total NADPH detection is as follows: glucose-6-phosphate (G6P) is oxidized to 6-phosphogluconate (6PG) by the action of glucose-6-phosphate dehydrogenase (G6PDH), during which NADP^+^ is reduced to NADPH. The generated NADPH, under the action of the electron mediator 1-Methoxy -5-methylphenazinium Methyl Sulfate (1-mPMS), reduces WST-8 into formazan that exhibits maximum absorption at around 450 nm. A proportional relationship exists between the amount of formazan formed in the reaction system and the total quantity of NADP^+^/NADPH in the sample.

The principle of free NADPH detection is as follows: after being heated in a 60°C water bath for 30 min, NADP^+^ in the sample decomposes while NADPH remains relatively stable. NADPH then reduces WST-8 into formazan, and the quantity of NADPH in the sample can be determined by colorimetric detection of the formed formazan.

## Results and Discussions

### Optimizing the Supply of Reducing Power in L-trp Producing Strains

**Fig. 2:**
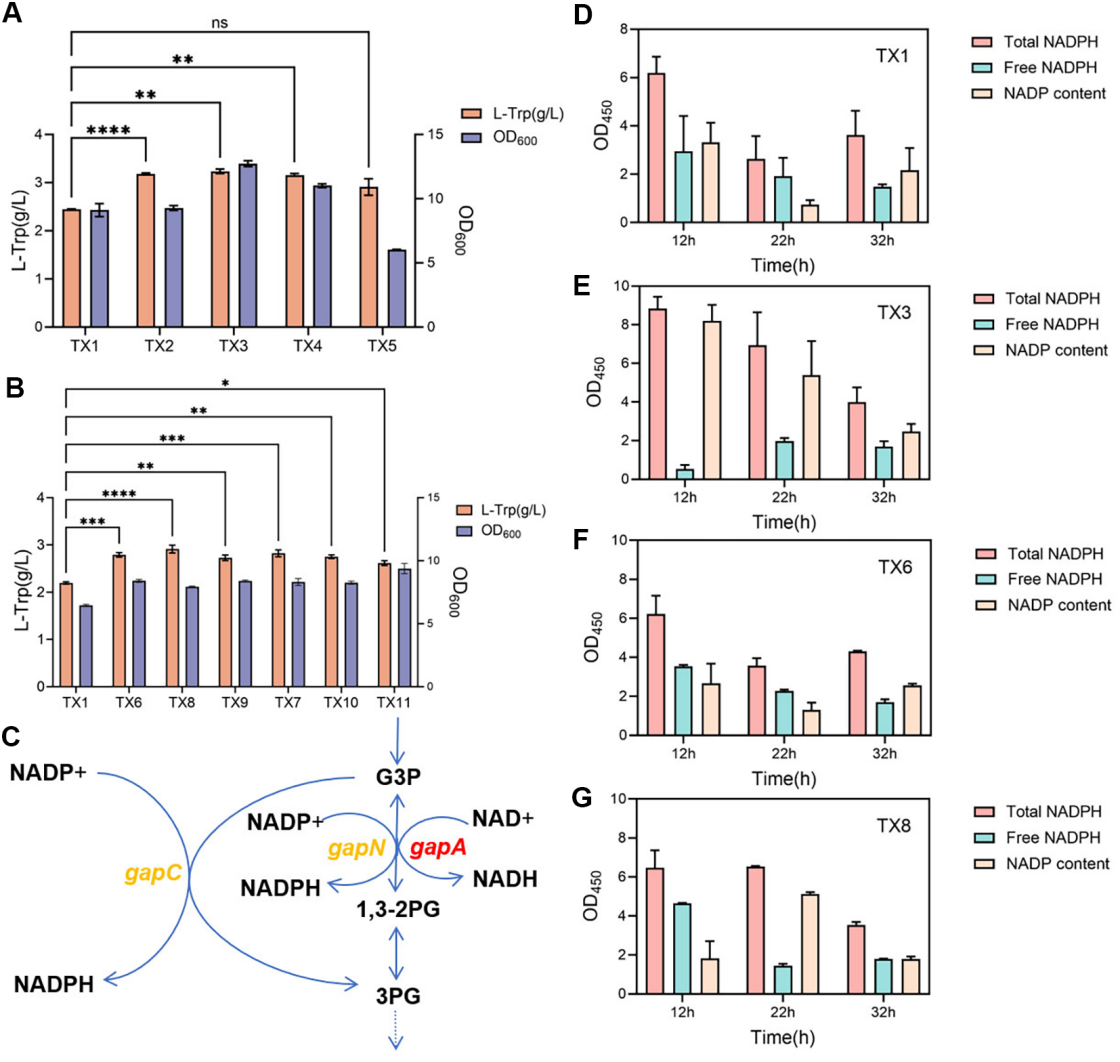
Reduction power engineering and determination of NADPH content. (**A**) Introduction of SthA mutants and wild-type genes, as well as PntAB mutants and wild-type genes, in strain TX3. (**B**) Introduction of the *gapC* gene from *Clostridium acetobutylicum* and the *gapN* gene from *Streptococcus mutans* into strain TX3, followed by proximal and distal attenuation of the *gapA* gene. (**C**) Metabolic reaction diagram involving *gapA*, *gapC*, and *gapN*. (**D**) Intracellular NADPH and NADP levels of strains TX1. (**E**) Intracellular NADPH and NADP levels of strains TX3. (**F**) Intracellular NADPH and NADP levels of strains TX6. (**G**) Intracellular NADPH and NADP levels of strains TX8. Experiments were conducted at least three times and the measurement results are presented as the means±SD. ns, **, *P* < 0.01, ****, *P* < 0.0001.

A multitude of studies have indicated that an imbalance in redox potential is often a pivotal factor that restricts the maximum yield of target fermentation products. In *E. coli*, the biosynthesis of tryptophan requires additional NADPH. NADPH not only acts as a reductant in the key reactions of tryptophan synthesis but also enhances the yield and production efficiency of tryptophan by maintaining redox balance and optimizing metabolic flux. In this study, site-directed mutagenesis was performed on the *sthA* and *pntAB* genes to obtain the *sthA* mutant (a single base substitution at alanine 145, changing it to valine) [32] and the *pntAB* mutant (a single base substitution at alanine 167, changing it to threonine) [32]. Subsequently, the wild-type and mutant *sthA* and *pntAB* genes were overexpressed in strain TX1, resulting in strains TX2, TX3, TX4, and TX5 (Table 1). Shake flask fermentation experiments were conducted to determine their effects on tryptophan production. The results showed that the expression of genes *sthA* or *pntAB* led to a significant increase in L-trp production (Fig. 2A). Among all variants, the SthA(A145V) mutant delivered the largest gain, boosting the L-tryptophan titer by 32.5% over the control strain TX1 (2.44 ± 0.01 g/l). SthA and PntAB are both enzymes that facilitate the conversion between NADH and NADPH, and they have been extensively utilized in recombinant bacteria for the production of compounds that rely on NADPH [33, 34]. Since L-tryptophan synthesis requires NADPH, enhancing the expression of SthA and PntAB effectively promotes the conversion from NADH to NADPH. The introduction of mutant SthA (A145V) enhances bacterial growth, which may be a key factor in boosting L-tryptophan production. As for why mutant SthA (A145V) improves bacterial growth, we speculate that its enhanced activity rapidly balances intracellular reducing power, reducing metabolic redox imbalance - induced damage to the bacteria.

To further enhance the supply of NADPH, as shown in Fig. 2C, genes related to NADPH synthesis were introduced into strain TX1, including glyceraldehyde-3-phosphate dehydrogenase (*gapC*) from *Clostridium acetobutylicum* [37], and glyceraldehyde 3-phosphate dehydrogenase (*gapN*) from *Streptococcus mutans* [39, 42, 43]. The *gapC* and *gapN* genes primarily function in the EMP (Embden-Meyerhof-Parnas) pathway. The *gapN* gene encodes an NADP^+^-dependent glyceraldehyde-3-phosphate dehydrogenase, which catalyzes the oxidation and phosphorylation of G3P to generate 1,3-bisphosphoglycerate while converting NADP^+^ to NADPH. The *gapC* gene encodes glyceraldehyde-3-phosphate dehydrogenase (GAPDH) in the EMP pathway, which catalyzes the glyceraldehyde-3-phosphate to produce 3-phosphoglycerate (3PG). Using CRISPR/Cas9 gene editing technology, the genes *gapN* and *gapC* were integrated into the genome of strain TX1 separately to obtain strains TX6 and TX7, respectively. Relative to the control strain TX1, TX6 (harboring *gapC*) and TX7 (harboring *gapN*) achieved 27% and 28.6%increases in tryptophan titer, respectively (Fig. 2B). These results indicate that the introduction of GapN or GapC enhances the tryptophan production capacity of the strains, but the effect is less pronounced than that of introducing the mutant SthA (A145V).

In an effort to further enhance the metabolic engineering effects of *gapC* or *gapN*, attempts were made to knock out the gene encoding glyceraldehyde-3-phosphate dehydrogenase (*gapA*) in *E. coli*. However, phenotypic analysis revealed that the *gapA* gene knockout mutant exhibited a cell lysis phenotype in LB medium and could not recover normal growth even after multiple subcultures, indicating that this gene plays an essential physiological role in the survival of the strain. Based on these findings, we employed dCas9-based technology to attenuate the expression of the *gapA* gene in strain TX6, resulting in the creation of strains TX8 and TX9. Similarly, attenuation of the *gapA* gene in strain TX7 led to the development of strains TX10 and TX11 (Table 1). Compared with strain TX6, proximal attenuation of *gapA* in TX8 further elevated the tryptophan titer by 4.5 %. In contrast, strains TX9, TX10, and TX11 exhibited no significant improvement or even a slight decrease in tryptophan production (Fig. 2B). Subsequent qPCR validation experiments confirmed the downregulation of the *gapA* gene, demonstrating that the dCas9-based attenuation technique effectively reduced its expression level (as shown in Fig. S1). The failure to enhance tryptophan production following the attenuation of *gapA* expression is likely due to the reduced generation of NADH, which severely disrupted the balance between NADH and NADPH. This disruption may have affected the expression of genes related to the pentose phosphate pathway [37, 41], leading to a decrease in the metabolic flux of this pathway and ultimately impacting the synthesis of tryptophan.

In this study, we observed a significant increase in tryptophan production in strains TX3 and TX8. Since the modifications in these strains primarily focused on balancing the supply of NADH and NADPH, we further conducted a quantitative analysis of intracellular NADPH levels. Fermentation was carried out for strains TX1, TX3, TX6, and TX8, and samples were taken at 12, 22, and 32 h. The NADPH content was measured using an NADPH assay kit provided by Biyuntian Biotechnology Co., Ltd. The specific experimental results are shown in Fig. 2D and 2E. At 12 h of fermentation, strain TX3, which overexpresses the *sthA* (A145V) gene, exhibited significantly higher levels of NADP^+^ and total NADPH compared to strain TX1, although the monomeric NADPH content showed a downward trend. This phenomenon can be attributed to the SthA (A145V) mutant, which causes a large amount of non-metabolically active NADPH to participate in tryptophan synthesis, leading to a higher rate of NADP^+^ generation, thereby accumulating a large amount of NADP^+^. This effect persisted until 22 h of fermentation but gradually weakened by 32 h. Compared to strain TX1, strain TX6, which carries the *gapC* gene, showed an overall increase in NADPH levels at different time points (Fig. 2F). After further attenuation of *gapA*, strain TX8 exhibited a significant increase in NADPH content at 12 h of fermentation compared to strain TX6 (Fig. 2G). At 22 h, the engineered strain TX8 had significantly higher total NADPH content and NADP^+^ accumulation than the control strain. This indicates that the introduction of the exogenous *gapC* gene and the attenuation of the endogenous *gapA* gene can promote the generation of more NADPH within the cell and enable it to more effectively participate in the tryptophan biosynthetic pathway, thereby enhancing tryptophan production. Notably, in the later stages of fermentation, the total NADPH content in all strains reached a low level, which may have affected the production and growth capabilities of the strains. Therefore, improving the reducing power levels in strains during the later stages of fermentation is key to efficient production of the target product throughout the entire process.

### Metabolomic Analysis of Strain TX3 and Identification of Metabolic Bottlenecks

To further explore the rate-limiting steps that restrict tryptophan production in strain TX3, a metabolomic analysis based on liquid chromatography-tandem mass spectrometry (LC-MS/MS) was employed to systematically analyze the metabolic changes in strain TX3 at different fermentation time points. Through comparative analysis, a total of 75 metabolites with significant differences were identified (Additional file: Table S2), which exhibited dynamic accumulation patterns during fermentation. Further functional annotation revealed that these differential metabolites were primarily distributed across the following key metabolic pathways: (1) EMP pathway; (2) pentose phosphate pathway; (3) shikimate pathway; and (4) tryptophan branch pathway.

**Fig. 3:**
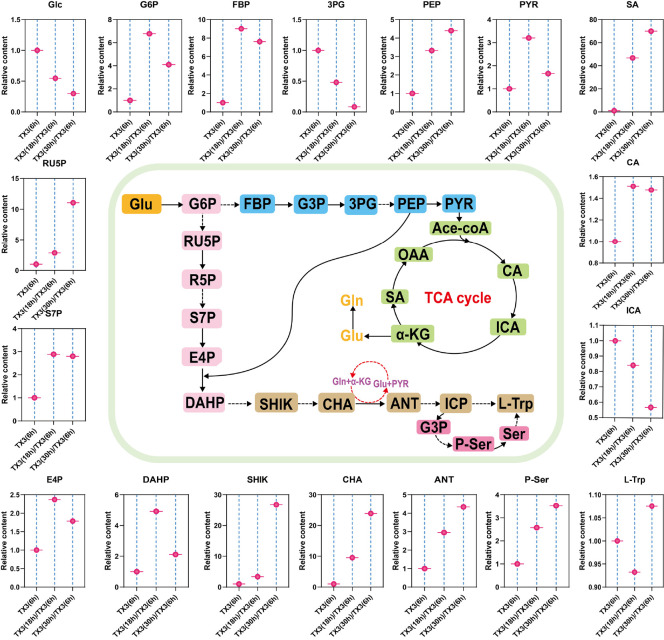
Relative abundance of metabolites in strain TX3. The metabolite levels at 6 hours in strain TX3 were used as the baseline, and the ratios were calculated to observe the changes in the relative abundance of metabolites.

Metabolomic analysis indicated that the TX3 mutant strain exhibited significant metabolic flux redistribution during fermentation (Fig. 3). Key metabolites of the EMP pathway, 3PG and PEP, displayed distinct accumulation patterns. The concentration of 3PG gradually decreases over time, while PEP continues to accumulate. To further determine the content of PEP, we measured the intracellular PEP levels in strain TX3 at 6, 18, and 30 h of fermentation using a PEP ELISA Detection Kit (Shanghai Kebio Biotechnology Co., Ltd., China). The results also showed that PEP concentrations increased progressively with fermentation time, reaching 12 nmol/g DCW at 30 h (see Fig. S2A). PEP can only enter the shikimate pathway after condensing with E4P to form DAHP. Therefore, we speculate that the insufficient supply of E4P may hinder the conversion of PEP, leading to the gradual accumulation of PEP. In addition, the trend of DAHP is similar to that of E4P, both of which first increase and then decrease with the progress of fermentation. This further confirms that E4P is the rate-limiting substrate for the synthesis of DAHP.

In the shikimate pathway, as the fermentation time extends, the accumulation of shikimic acid and chorismic acid shows significant changes. Specifically, compared to the 6-h fermentation time, the difference in accumulation multiples of shikimic acid and chorismic acid reaches its maximum at the 30-h fermentation mark. To determine the specific accumulation amount of shikimic acid, we analyzed the shikimic acid levels in the fermentation supernatant at 6 h, 18 h, and 30 h via HPLC. The extracellular concentration of shikimic acid increased steadily from 0.35 mg/l to 11.8 mg/l as fermentation progressed (see Fig. S2B). This phenomenon is likely due to the presence of rate-limiting enzymes in the shikimate pathway. The existence of rate-limiting enzymes may cause the metabolic flux to be restricted at certain key steps, leading to a large accumulation of metabolic intermediates before these steps. For example, certain enzymes in the shikimate pathway may have insufficient activity or be subject to feedback inhibition, preventing shikimic acid and chorismic acid from being further metabolized in a timely manner, thereby accumulating within the cells. This accumulation not only reflects the regulatory mechanisms of the metabolic pathway but may also have a significant impact on the metabolic balance of the cells.

In the tryptophan branch pathway, both anthranilate and phosphoserine show an accumulation trend during fermentation. However, the difference in their accumulation multiples is not as significant as that of shikimic acid and chorismic acid. Although non-targeted metabolomics cannot achieve absolute quantification of metabolites, detecting their presence indicates that anthranilate and phosphoserine do accumulate during fermentation. While we cannot determine whether this accumulation is statistically significant, it is clear that they continue to accumulate over time. Therefore, it is possible to enhance the metabolic flux of the tryptophan branch pathway to ensure the full conversion of anthranilate and phosphoserine.

### Relieving Rate-Limiting Steps in Metabolic Pathways

**Fig. 4:**
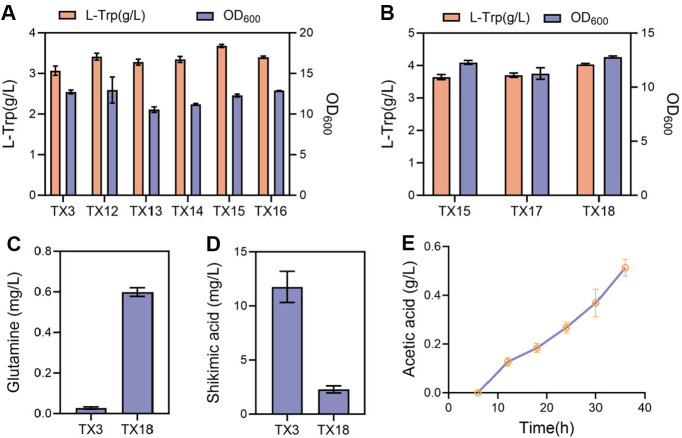
Effects of expanding the shikimate pathway, expanding the tryptophan branch pathway, and enriching E4P pool on L-trp biosynthesis. (**A**) Influence of GlnA mutant, *aroK*, *aroL*, *aroA* and *aroC* enhancement on Ltrp biosynthesis and cell growth. (**B**) Influence of *trpB* enhancement and effects of increased E4P supply on L-trp production. (**C**) The content of glutamine in the fermentation broth of strains TX3 and TX18. (**D**) The content of shikimic acid in the fermentation broth of strains TX3 and TX18. (**E**) The accumulation of acetic acid during the fermentation process of strain TX18. Experiments were conducted at least three times and the measurement results are presented as the means ± SD.

As fermentation progresses, the significant difference in the accumulation multiples of shikimate and chorismate suggests that the shikimate pathway is the most rate-limiting. Chorismate can be converted to anthranilate in the presence of glutamine, which donates an amino group, catalyzed by anthranilate synthase. Glutamine itself is synthesized from glutamate by the action of glutamine synthetase (GlnA). Given that the genes encoding anthranilate synthase (*trpE*^fbr^*D*) are already overexpressed on the plasmid, to enhance the flux of chorismate into the downstream tryptophan branch pathway, we focused on improving the efficiency of glutamate conversion to glutamine. The expression of *glnA* in *E. coli* is under complex regulation, involving two tandem promoters (*glnApl* and *glnAp2*). The upstream promoter *glnApl* requires the catabolite activating protein for expression and is repressed by nitrogen regulator I (NR_I_), which is the product of the *glnG* gene. The downstream promoter *glnAp2* requires both NR_I_ and the product of the *glnF* gene, and its full expression is contingent upon growth under nitrogen-limited conditions [44, 45]. To enhance the conversion of glutamate to glutamine, we introduced a mutant GlnA (Y405F) from *C. glutamicum* [46, 47] resulting in strain TX12. In *C. glutamicum*, GlnA is reversibly inactivated by adenylylation of Tyr405 under high ammonium, and replacing this residue with Phe (Y405F) renders the enzyme insensitive to ammonium control. Compared to strain TX3, this modification led to an 11.2%increase in tryptophan production, indicating that enhancing the supply of glutamine can promote the conversion of chorismate into the tryptophan synthesis pathway, thereby facilitating the production of tryptophan. Based on this, GltB and GltD can catalyze the conversion of 2-oxoglutarate and glutamine into glutamate. To increase the supply of glutamine, it is also possible to consider weakening or knocking out the *gltB* or *gltD* gene in the future. Shikimate is converted to chorismate through a three-step process catalyzed by shikimate kinase (AroK/AroL), 3-phosphoshikimate 1-carboxyvinyltransferase (AroA), and chorismate synthase (AroC). To further optimize L-tryptophan production, we introduced additional copies of *aroK*, *aroL*, *aroA*, and *aroC* into the *yjiT* locus of the genome of strain TX12, generating strains TX13, TX14, TX15, and TX16, respectively. The results showed that increasing the copy number of the *aroA* gene significantly enhanced L-tryptophan synthesis, boosting production by 19.8% relative to strain TX3. However, increasing the copy numbers of the *aroK*, *aroL*, or aroC genes had no significant impact on L-tryptophan production (Fig. 4A). The enzyme encoded by *aroA* catalyzes the conversion of shikimate 3-phosphate and PEP into 5-enolpyruvoyl-shikimate 3-phosphate [49, 50]. We speculate that the accumulation of shikimate resulted from the AroA enzyme's inability to promptly convert shikimate 3-phosphate into downstream products. Moving forward, in the future, we can consider introducing an exogenous AroA enzyme or engineering the AroA enzyme to enhance its activity, which may further increase tryptophan production.

Tryptophan is synthesized from the substrates indole and serine, catalyzed by the enzyme tryptophan synthase (TrpB) [51]. However, both indole and serine are toxic to cells to some extent. Metabolomics analysis revealed that the accumulation of anthranilate (the precursor of indole) and phosphoserine (the direct precursor of serine) to some extent. We speculate that this accumulation is likely due to the low catalytic efficiency of TrpB [52]. The enzyme TrpB is unable to rapidly convert indole and serine into tryptophan, thereby impeding the metabolic flux. Meanwhile, due to the toxicity of indole and serine, cells cannot accumulate large amounts of these two substances, which in turn leads to the accumulation of their precursors-anthranilate and phosphoserine. To address this issue, we optimized the tryptophan operon on the plasmid by adding an additional tac promoter in front of the *trpB* gene, resulting in the construction of strain TX17. Relative to strain TX15, the tryptophan titer of TX17 increased by only 1.6 % - a change that is not statistically significant (Fig. 4B). This suggests that although the expression of the *trpB* gene has been enhanced, further optimization of other relevant factors may be needed to achieve a more significant increase in production.

Metabolomics analysis identified the supply of E4P as a rate-limiting step in the tryptophan synthesis pathway. Previous studies have demonstrated that the *xfp* gene, which encodes phosphoketolase, is more effective than the *tktA* gene in redirecting carbon flux from fructose-6-phosphate (F6P) toward E4P formation. This redirection significantly enhances the production of aromatic derivatives in yeast [53]. To boost the supply of E4P, we introduced phosphoketolase from *Bifidobacterium adolescentis* into our system [53]. Specifically, we overexpressed the *xfp* gene in *ackA* locus of strain TX15, resulting in the creation of strain TX18. As a result, the tryptophan production in strain TX18 increased by 10.8% relative to TX15 (Fig. 4B). This result indicates that increasing the supply of E4P effectively promotes the combination of accumulated PEP with E4P, thereby facilitating their entry into the shikimate pathway. This process not only enhances the metabolic flux of the shikimate pathway but also leads to an increase in tryptophan production. This finding further confirms the crucial role of the shikimate pathway in aromatic amino acids synthesis [54] and also provides an important theoretical basis for optimizing tryptophan production through metabolic engineering.

The increased supply of glutamine (from 0.03 mg/l to 0.6 mg/l, Fig. 4C) enhanced the conversion of chorismate to anthranilate, thereby boosting the production of tryptophan. Overexpression of the downstream shikimate pathway genes *aroK*, *aroL*, *aroA*, and *aroC* all had varying impacts on cell growth. However, only the overexpression of *aroA* significantly increased tryptophan production. Analysis of the supernatant after fermentation of strains TX3 and TX18 revealed that the concentration of shikimic acid decreased from 11.7 mg/l to 2.3 mg/l (Fig. 4D). During fermentation, although acetic acid accumulated continuously, its overall concentration remained low (Fig. 4E). This suggests that the overexpression of the *aroA* and *glnA* (Y405F) genes redirected the metabolic flux of shikimate and chorismate, favoring the tryptophan pathway over by-product formation. This strategic redirection significantly enhanced the conversion efficiency of shikimate and chorismate to downstream products. By efficiently channeling the accumulated shikimate and chorismate into the tryptophan synthesis pathway, this approach not only mitigated their intracellular accumulation but also enhanced their utilization in tryptophan production. Collectively, these findings underscore the pivotal role of *aroA* and *glnA* (Y405F) in guiding the metabolic flux of the shikimate pathway towards tryptophan synthesis. Increasing the expression of *trpB* had no effect on the accumulation of anthranilate and phosphoserine. Subsequently, we attempted to increase the copy number of the entire tryptophan operon, but this also failed to boost tryptophan production. This lack of improvement may be due to the relatively low accumulation levels of anthranilate and phosphoserine. Therefore, future work could focus on quantitative analysis of metabolites in the tryptophan branch pathway and attempt to modify the TrpB enzyme to enhance its activity or introduce an exogenous TrpB enzyme that can directly utilize phosphoserine [55], to see if this can alleviate the accumulation of precursors.

### Optimization of Shake Flask Fermentation Medium

**Fig. 5:**
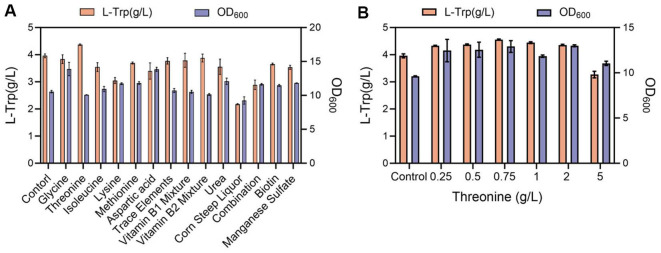
The impact of flask fermentation medium optimization on L-trp production. (**A**) Impact of adding various nutritional components to the fermentation medium on L-trp production. Listed from left to right: control group, addition of glycine, threonine, isoleucine, lysine, methionine and aspartic acid, addition of trace elements, vitamin B1 mix, vitamin B2 mix, urea, corn steep liquor, combination (0.5 g/l glycine, 0.5 g/l threonine, 0.5 g/l isoleucine, 0.5 g/l lysine, 0.5 g/l methionine, 0.5 g/l aspartic acid, 0.002 g/l biotin, 2 ml/l VB2 mix, 2 ml/l trace elements), biotin, and manganese sulfate. (**D**) Effects of varying threonine concentrations in flask fermentation medium on L-trp production. Experiments were conducted at least three times and the measurement results are presented as the means ± SD.

Different strains exhibit distinct requirements for the components in the fermentation medium. To fully unlock the tryptophan production potential of strain TX18, we have optimized the fermentation medium. The amino acid composition of the fermentation medium was supplemented and optimized, including glycine, threonine, isoleucine, lysine, methionine, and aspartic acid. Additionally, trace elements, vitamins, and various organic nitrogen were added to the fermentation medium to evaluate their impact on tryptophan production. The fermentation conditions were set for single-factor addition experiments, with the final exogenous addition concentrations as follows: 0.5 g/l glycine, 0.5 g/l threonine, 0.5 g/l isoleucine, 0.5 g/l lysine, 0.5 g/l methionine, 0.5 g/l aspartic acid, 2 ml/l trace elements (MnSO_4_·H_2_O 4.5 g/l, ZnSO_4_ 6.4 g/l, Co(NO_3_)_2_·6H_2_O, CuSO_4_·5H_2_O), 2 ml/l Vitamin B mixture 1 (VB1, VB3, VB5, VB12, 1 g/l each), 2 ml/l Vitamin B mixture 2 (VB1, VB3, VB5, VB6, 1 g/l each), 1.5 g/l urea, 5 g/l corn steep liquor, combination (0.5 g/l glycine, 0.5 g/l threonine, 0.5 g/l isoleucine, 0.5 g/l lysine, 0.5 g/l methionine, 0.5 g/l aspartic acid, 0.002 g/l biotin, 2 ml/l VB2 mix, 2 ml/l trace elements), 0.002 g/l biotin, 0.02 g/l manganese sulfate (Fig. 5A).

The results indicated that the addition of threonine significantly enhanced tryptophan production (Fig. 5A). Therefore, a gradient optimization experiment for threonine concentration was conducted, testing threonine additions of 0.25 g/l, 0.5 g/l, 0.75 g/l, 1 g/l, 2 g/l, and 5 g/l. The experimental results showed that when the threonine concentration was 0.75 g/l, the tryptophan production of the strain reached its highest value, at 4.56 ± 0.015 g/l (Fig. 5B).

Reports have indicated that the biosynthesis of L-threonine and L-isoleucine is impeded by L-serine [56, 57] This inhibition occurs because L-serine suppresses the activity of threonine dehydrogenase I (ThrA) [58], a pivotal enzyme for L-threonine production. Given that L-serine serves as a direct precursor for L-tryptophan and is converted to it by the TrpB enzyme, its presence may downregulate ThrA expression. This could lead to deficiencies in L-threonine and L-isoleucine, creating an amino acid shortfall that hampers cell growth and, consequently, reduces L-tryptophan yield. However, when we supplemented the medium with L-isoleucine, tryptophan production did not rise. This might be because L-threonine can be metabolized into L-isoleucine by IlvA, IlvC, IlvE, and IlvD enzymes. This conversion could exacerbate the L-threonine deficiency while L-isoleucine meets cellular demands. To test our hypothesis, we fermented strain TX18 in a medium devoid of L-threonine and sampled at 6, 18, and 30 h. After disrupting the samples with ultrasonication, we quantified intracellular amino acids using an amino acid analyzer. The data revealed a steady increase in intracellular tryptophan (Fig. S3B), while L-threonine was undetectable at all time points (Fig. S3A). Other amino acids were present, with L-isoleucine accumulating at 6 and 18 h. These findings underscore the L-threonine deficiency. Thus, adding L-threonine to the medium effectively boosts L-tryptophan production by addressing this shortfall. We have included these details in the supplementary materials.

Typically, optimizing the culture medium can significantly enhance the yield of the target product. By fine-tuning the concentration of threonine, the tryptophan production of strain TX18 increased significantly by 15.1%compared with that without threonine addition, reaching a yield of 18.4%, which is equivalent to 81% of the theoretical conversion rate. This rate surpasses the 16.7% yield obtained in our mutagenized strains [5], further underscoring the advantages of using strains with a clear genetic background for subsequent modifications. However, this conversion rate remains lower than the 22.7% achieved by knocking out the phosphotransferase system (PTS) [59]. This suggests that while significant progress has been made with strain TX18, there is still potential for further enhancement. Future work could focus on replacing the PTS system with alternative glucose uptake systems in strain TX18 to further boost its tryptophan conversion rate and advance the industrial production of tryptophan.

## Conclusion

Microbial fermentation, as a green, efficient, and sustainable bioproduction technology, has garnered significant attention in the field of biosynthesis in recent years. Through a series of metabolic engineering strategies, including enhancing the supply of intracellular reducing power, pinpointing rate-limiting steps via metabolomics analysis, and optimizing culture medium formulations, we have successfully developed a high-performance tryptophan-producing strain. This strain not only has a clear genetic background, facilitating subsequent research and modification, but also achieved a remarkable breakthrough in tryptophan production, increasing it by 86.6%compared to the original level. These achievements not only provide important theoretical basis for in-depth studies on the tryptophan biosynthetic pathway but also lay a solid foundation for the future development of more efficient tryptophan-producing strains, holding significant scientific and practical value.

## Supplemental Materials

Supplementary data for this paper are available on-line only at http://jmb.or.kr.



## Figures and Tables

**Table 1:** Strains used in this study.