# Supplementary material for: Optimizing L-Tryptophan Production in Escherichia coli through Redox Balancing and Metabolomics Analysis
Source: J Microbiol Biotechnol. 2025 Dec 29;35:e2508025. doi: 10.4014/jmb.2508.08025 (PMC12790993; doi:10.4014/jmb.2508.08025)
Supplement: Supplementary file 1 [file jmb-35-e2508025-supple.pdf]

J. Microbiol. Biotechnol. 2025. 35: e2508025
https://doi.org/10.4014/jmb.2508.08025

Supplementary Figures and Tables

Optimizing L-Tryptophan Production in *Escherichia coli* through Redox Balancing and Metabolomics Analysis

Tongxin Wan[1,2†], Dongqin Ding[2,3,5†], Junqing Chen[2,6], Yaru Zhu[2,5], Huiying Wang[2,3,5], Zixiang Xu[2,5], Junlin Yang[7], Yufeng Wang[7], Jia Song[1*], Dawei Zhang[2,3,4,5*]

[1] College of Biotechnology, Tianjin University of Science & Technology, Tianjin 300457, China.

[2] Tianjin Institute of Industrial Biotechnology, Chinese Academy of Sciences, Tianjin 300308, China.

[3] University of Chinese Academy of Sciences, Beijing 100049, China.

[4] State Key Laboratory of Engineering Biology for Low-Carbon Manufacturing, Tianjin Institute of Industrial Biotechnology, Chinese Academy of Sciences, Tianjin 300308, China.

[5] National Center of Technology Innovation for Synthetic Biology, Tianjin 300308, China.

[6] Dalian Polytechnic University, Dalian, 116000, PR China

[7] Yuxing Biotechnology (GROUP) CO., LTD.

* Jia Song, * Dawei Zhang.

E-mail: tjsongjia@tust.edu.cn, zhang_dw@tib.cas.cn

Authors' contributions

†These two authors contributed equally and share the first authorship.

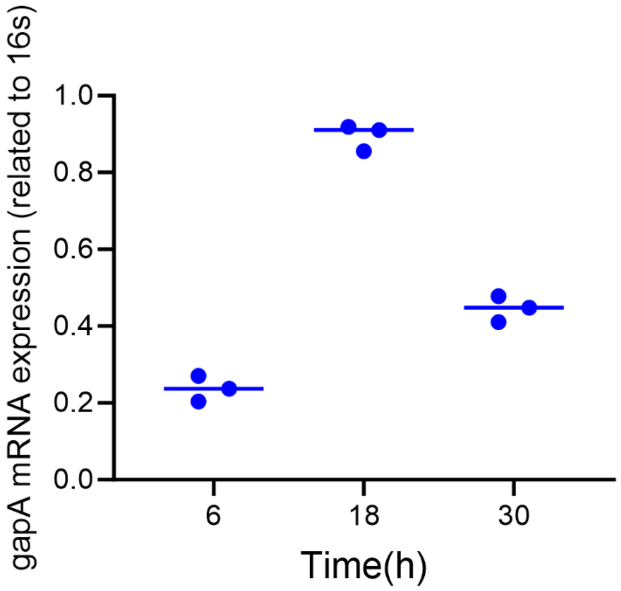

Fig. S1. The qPCR data of strains TX6 and TX8 with a weakened *gapA* promoter. Experiments were conducted at least three times and the measurement results are presented as the means±SD.

Fig. S2

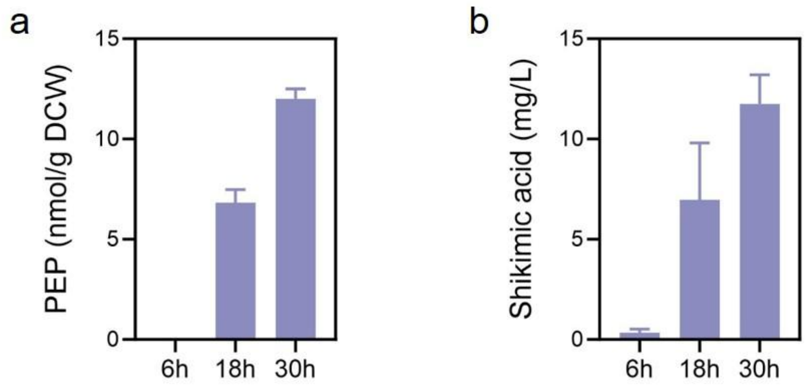

Fig. S2. Determination of the contents of intermediate metabolites PEP and shikimic acid in the L-tryptophan biosynthetic pathway (a) Intracellular PEP content in strain TX3 at 6, 18, and 30 hours of fermentation. (b) Extracellular shikimic acid content in strain TX3 at 6, 18, and 30 h of fermentation. Experiments were conducted at least three times and the measurement results are presented as the means±SD.

Fig. S3

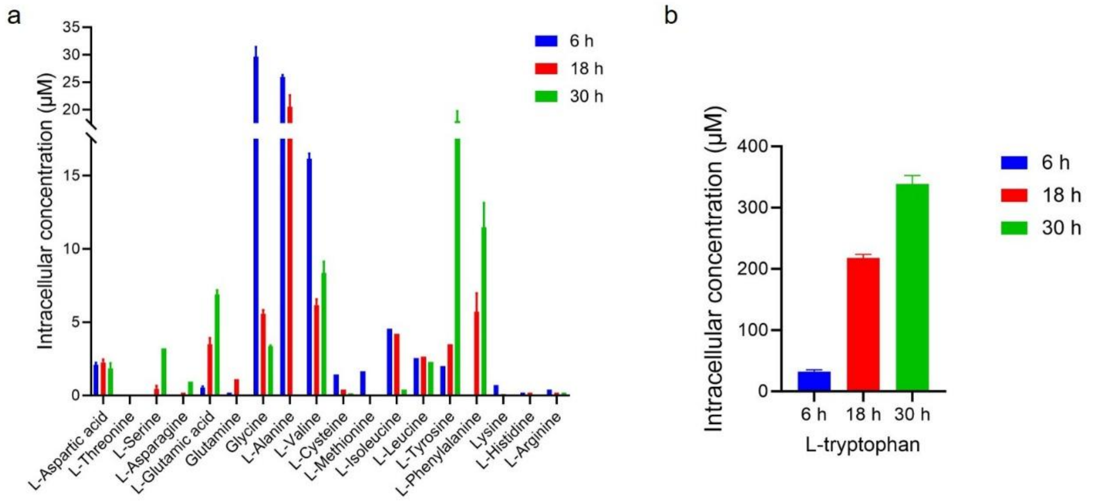

Fig. S3. The intracellular amino acid content of strain TX18 was quantitatively analyzed at 6, 18, and 30 h of fermentation using an amino acid analyzer. This analysis included: a. Determination of intracellular levels of L-aspartic acid, L-threonine, L-serine, L-asparagine, L-glutamic acid, glutamine, glycine, L-alanine, L-valine, L-cysteine, L-methionine, L-tyrosine, L-phenylalanine, lysine, L-histidine, and L-arginine; b. Determination of intracellular L-tryptophan levels. Experiments were conducted at least three times and the measurement results are presented as the means ±SD.

Table S1. Primers used in this study.

Primer	Sequence (5’→3’)
**Primers for sensor construction**	
ycjV-up-F	GAAGAATCCATGGGCCTGTGCTCTATGTCCACTCACTACGC
ycjV-down-R	GAGAATCCAAGCTTCCATTCATCCACGTTTACCCGCGCTAT
ycjV-ver-F	TAGCGCGGGTAAACGTGGATGAATGGAAGCTTGGATTCTCAC
ycjV-ver-R	CGTAGTGAGTGGACATAGAGCACAGGCCCATGGATTCTTCG
ycjV-N20-F	CAGATCTTAGCCAACGTCAGCGAGTGGCGCTGTTTTAGAGCTAGAAATAGC
ycjV-N20-R	CTCTAAAACAGCGCCACTCGCTGACGTTGGCTAAGATCTGACTCCATAAC
sthA-F	TGTGAGCGGATAACAATTCCCCAATAATTTTGTTTAACTTTAAGAAGGAGATATACATATGCCACATTCCTACGATTACGA
sthA-R	TGTTATTTTCCTCTTTAAAACAGGCGGTTTAAACCG
sthA-down-F	GTTTAAACCGCCTGTTTTAAAGAGGAAAATAACATTTCCGCC
sthA-up-R	GTTAAACAAAATTATTGGGGAATTGTTATCCGCTCACAATTCCACACATTATACGAGCCGATGATTAATTGTCAAGTCCTTCACCACATGCACCT
pntAB-F	GGTGCATGTGGTGAAGGACAAGTAGTGATTCGTGCCGGG
pntAB-R	GAAATGTTATTTTCCTCTTTACAGAGCTTTCAGGATTGCATCC
pntAB-down-F	GATGCAATCCTGAAAGCTCTGTAAAGAGGAAAATAACATTTCCGCC

pntAB-up-R	CCGGCACGAATCACTACTTGTCCTTCACCACATGCACCT
pflB-up-F	GACGAAGAATCCATGGGCCTGTATGGTGCTGCCGGTCGCGAT
pflB-down-R	GGTGAGAATCCAAGCTTCCATTCACATGCGTGTCCCAGGTGTC
pflB-ver-F	GACACGCATGTGAATGGAAGCTTGGATTCTCACCAATAAAAAAC
pflB-ver-R	CCGGCAGCACCATACAGGCCCATGGATTCTTCGTCTGTTT
gapC/N-up-R	TTACGCAGCGTCAAGCGGAATCGTGTCAATCATTGCGACAAATCAATCCTGTGCCTAAGCATTACGCGACTGAATTTACTGCGTACTTCGACAACC
gapC-F	CCGCTTGACGCTGCGTAAGGTTTTTGTAATTTTACAGGCAACCTTTTATTCACTAACAAATAGCTGGTGGAATATATGGCTAAAATCGCTATCAACG
gapC-R	ACGATTTCAGTCAAATCTAATTATTTAGCGATTTTAGCGAAGTAAGCCAGGGTAC
gapC-down-F	CTGGCTTACTTCGCTAAAATCGCTAAATAATTAGATTTGACTGAAATCGTACAGTAAAAAGCG
pflB-N20-F	CGAGGGTGACGAGTCCTTCCGTTTTAGAGCTAGAAATAGCAAGTTAAAATAAGGCTAGTC
pflB-N20-R	GCTCTAAAACGGAAGGACTCGTCACCCTCGGCTAAGATCTGACTCCATAACAGAGTACTCG

gapN-F	CCGCTTGACGCTGCGTAAGGTTTTTGTAATTTTACAGGCAACCTTTTATTCACTAACAAATAGCTGGTGGAATATATGACCAAACAGTACAAAAACTAC
gapN-R	ACGATTTCAGTCAAATCTAATTATTTGATGTCGAAAACAACAGATTTAACGGTG
gapN-ver-F	TGTTGTTTTCGACATCAAATAATTAGATTTGACTGAAATCGTACAGTAAAAAGCG
distal attenuation-F	AAATGTTACCGCTGAACGTGATCGTTTTAGAGCTAGAAATAG
distal attenuation-R	AACGATCACGTTCAGCGGTAACATTTTCTCTATCACTGATAGG
proximal attenuation-F	GAAAACCTGTTAGACGCTGATTACAGTTTTAGAGCTAGAAATAGC
proximal attenuation-R	CATGTATGCCATGTAATCAGCGTCTAACAGGTCGTTGATTGCAACG
glnA-up-F	AAGAATCCATGGGCCTGTAACTTTGCCTCAGGCATTAG
glnA-up-R	GGAATTGTTATCCGCTCACAATTCCACACATTATACGAGCCGATGATTAATTGTCAAACTTTAACTCTCCTGGATTG
glnA405-F	TAATGTGTGGAATTGTGAGCGGATAACAATTCCCCAATAATTTTGTTTAACTTTAAGAAGGAGATATACATGTGGCGTTTGAAACCCCGGA
glnA405-R	CACGGCAACTAAAACACTTAGCAGTCGAAGTACAATTC
glnA-down-F	TGTACTTCGACTGCTAAGTGTTTTAGTTGCCGTGGAAAC
glnA-down-R	GAATCCAAGCTTCCAACCGTCGCCAGGTTGTCATCGATT

glnA-ver-F	TCGATGACAACCTGGCGACGGTTGGAAGCTTGGATTCTC
glnA-ver-R	TGCCTGAGGCAAAGTTACAGGCCCATGGATTCTTCG
glnA-N20-F	CTTAGCCACAACGTAGCGCACCGCTTGTTTTAGAGCTAGAAATAGC
glnA-N20-R	CTAAAACAAGCGGTGCGCTACGTTGTGGCTAAGATCTGACTCCATAAC
yjiT-up-F	GAATCCATGGGCCTGTTAAATGTCAGCTACTGAATACTTTTTG
yjiT-down-R	GAGAATCCAAGCTTCCATTCAATAGCCAAAGGGCCAGCCTT
yjiT-up-R	CAAAATTATTGGGGAATTGTTATCCGCTCACAATTCCACACATTATACGAGCCGATGATTAATTGTCAAGTAGCTGCGTTAATTGGGTATA
yjiT-ver-F	CTGGCCCTTTGGCTATTGAATGGAAGCTTGGATTCTCAC
yjiT-ver-R	AGTAGCTGACATTTAACAGGCCCATGGATTCTTCG
yjiT-N20-F	CAGATCTTAGCGCTATGCGCAGCCTCTGTAGGTTTTAGAGCTAGAAATAGC
yjiT-N20-R	CTAAAACCTACAGAGGCTGCGCATAGCGCTAAGATCTGACTCCATAACAGAG
aroK-F	TGTGAGCGGATAACAATTCCCCAATAATTTTGTTTAACTTTAAGAAGGAGATATACATATGGCAGAGAAACGCAATAT
aroK-R	GTAACTGTAACGTCCTTTAGTTGCTTTCCAGCATGT
aroK-down-F	GCTGGAAAGCAACTAAAGGACGTTACAGTTACAGCA
aroL-F	TGTGAGCGGATAACAATTCCCCAATAATTTTGTTTAACTTTAAGAAGGAGATATACATATGACACAACCTCTTTTTCTGATC

aroL-R	GTAACTGTAACGTCCTTCAACAATTGATCGTCTGTG
aroL-down-F	GACGATCAATTGTTGAAGGACGTTACAGTTACAGCA
aroA-F	TGTGAGCGGATAACAATTCCCCAATAATTTTGTTTAACTTTAA GAAGGAGATATACATATGGAATCCCTGACGTTACAA
aroA-R	GTAACTGTAACGTCCTTCAGGCTGCCTGGCTAATCC
aroA-down-F	TAGCCAGGCAGCCTGAAGGACGTTACAGTTACAGCA
aroC-F	TGTGAGCGGATAACAATTCCCCAATAATTTTGTTTAACTTTAA GAAGGAGATATACATATGGCTGGAAACACAATTGG
aroC-R	GTAACTGTAACGTCCTTTACCAGCGTGGAATATCAG
aroC-down-F	TATTCCACGCTGGTAAAGGACGTTACAGTTACAGCA
tac-trpB-F	TGTGAGCGGATAACAATTCCCCAATAATTTTGTTTAACTTTAA GAAGGAGATATACATATGACAACATTACTTAACCCC
tac-trpB-R	CAAAATTATTGGGGAATTGTTATCCGCTCACAATTCCACACAT TATACGAGCCGATGATTAATTGTCAATTAATATGCGCGCAGCG TCTGG
Baxfp-up-F	GAAGAATCCATGGGCCTGTGTAATGCTGTCCCCGGCGAA
Baxfp-up-R	TTGGGGAATTGTTATCCGCTCACAATTCCACACATTATACGAG CCGATGATTAATTGTCAAGGAAGTACCTATAATTGATACGTGG C

Baxfp-F	GTATAATGTGTGGAATTGTGAGCGGATAACAATTCCCCAATAATTTTGTTTAACTTTAAGAAGGAGATATACATATGACGAGCCCGGTGATTGG
Baxfp-R	TGACCAGACCTTCCAGCGTTATTCGTTATCGCCCGCGGTCG
Baxfp-down-F	GACCGCGGGCGATAACGAATAACGCTGGAAGGTCTGGTCATG
Baxfp-down-R	AGAATCCAAGCTTCCATTCACCGAAACGTGCAGCCAGGTTG
Baxfp-ver-F	AACCTGGCTGCACGTTTCGGTGAATGGAAGCTTGGATTCTCAC
Baxfp-ver-R	TCGCCGGGGACAGCATTACACAGGCCCATGGATTCTTCG
Baxfp-N20-F	TCTTAGCCACAACCCGGCTCACCTGATGTTTTAGAGCTAGAAATAGCAAGTTAAAATAAGG
Baxfp-N20-R	CTAGCTCTAAAACATCAGGTGAGCCGGGTTGTGGCTAAGATCTGACTCCATAACAGAG
